# Ergot and Ergot Alkaloids in Cereal Grains Intended for Animal Feeding Collected in Slovenia: Occurrence, Pattern and Correlations

**DOI:** 10.3390/toxins12110730

**Published:** 2020-11-21

**Authors:** Janja Babič, Gabrijela Tavčar-Kalcher, Franci Aco Celar, Katarina Kos, Matjaž Červek, Breda Jakovac-Strajn

**Affiliations:** 1 Institute of Food Safety, Feed and Environment, Veterinary Faculty, University of Ljubljana, Gerbičeva 60, 1000 Ljubljana, Slovenia; gabrijela.tavcar-kalcher@vf.uni-lj.si (G.T.-K.); Breda.JakovacStrajn@vf.uni-lj.si (B.J.-S.); 2 Department of Agronomy, Biotechnical Faculty, University of Ljubljana, Jamnikarjeva 101, 1000 Ljubljana, Slovenia; Franc.Celar@bf.uni-lj.si (F.A.C.); katarina.kos@bf.uni-lj.si (K.K.); 3 Emona RCP, Kavčičeva ulica 72, 1000 Ljubljana, Slovenia; matjaz.cervek@e-rcp.si

**Keywords:** ergot alkaloid occurrence, sclerotia, cereals, LC-MS/MS, correlation, survey

## Abstract

This four-year study reports the occurrence of ergot alkaloids (EAs) in cereals intended for animal feeding collected in Slovenia. A total of 517 samples of cereals were analysed using liquid chromatography-tandem mass spectrometry for the presence of EAs. The sample set included wheat, rye, triticale, oat, spelt and barley. The study revealed that 17% of the analysed cereal samples were contaminated with at least one ergot alkaloid. EAs have two epimeric forms: -ine and -inine. The incidence rates of the -ine and -inine forms in the analysed samples were 16% and 15%, respectively. The highest contamination rates were observed in rye (54%), oat (50%) and spelt (30%), where the highest mean concentrations of total EAs were also determined (502 µg/kg, 594 µg/kg and 715 µg/kg, respectively). However, the highest concentrations of total EAs were found in wheat and rye (4217 µg/kg and 4114 µg/kg, respectively). The predominant EAs were ergometrine, ergosine and ergocristinine. The occurrence of six or more ergot alkaloids was observed in 49% of the positive samples. A weak correlation (*p* = 0.284) in the positive samples was found between the mass of sclerotia and the total concentrations of EAs using the Spearman correlation coefficient.

## 1. Introduction

**Figure 1:**
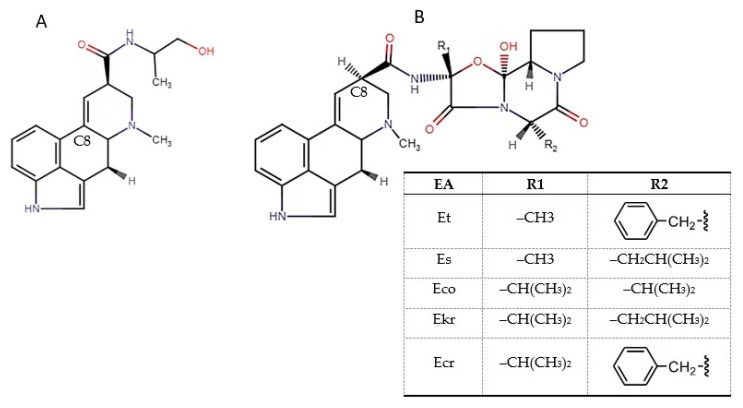
Chemical structures of EAs. (**A**) Ergometrine—Em and (**B**) ergotamine—Et, ergosine—Es, ergocornine—Eco, ergokriptine—Ekr and ergocrystine—Ecr.

Ergot alkaloids (EAs) are secondary metabolites produced by fungi of the genus *Claviceps*. In Europe, *Claviceps purpurea* is the most widespread *Claviceps* species. The term “*purpurea*” comes from its ability to replace kernels in cereals with hard purple-coloured ergot bodies (sclerotia) containing a variety of alkaloids. *Claviceps purpurea* is known to cause more than 400 plant species, including grasses and economically important cereal grains, such as rye, wheat, triticale, barley, millet and oats, to be infected with the disease known as ergot [1,2,3]. Sclerotia of *C. purpurea* vary in size (2–20 mm) and are up to ten-fold larger than a normal grain. They have a hard, protective, and black to dark purple cortex outside and a white to grey inside medulla. Sclerotia contain various classes of EAs. More than 50 EAs have been identified [1,4], the most prominent of which are ergometrine (Em), ergotamine (Et), ergosine (Es), ergocristine (Ecr), ergocryptine (Ekr) and ergocornine (Eco), as well as their epimeric forms (-inines), which are biologically less active, but can be converted to the -ine form under various conditions [1,5]. EAs are characterized by the presence of a tetracyclic ergoline ring system and can be classified into four major groups based on the substitution at C^8^ [4]: clavine alkaloids and 6,7-secoergolenes, simple lysergic acid derivatives (Em; Figure 1A), ergopeptine alkaloids (Es, Eco, Ekr, Ecr; Figure 1B), and ergopeptam alkaloids (Et, Figure 1B). The chemical structures of the epimeric forms -inine are the same as of the main EAs, but with different configuration at C^8^, which is the centre of symmetry. The main EAs are left-hand rotation (R)-isomers, but their epimeric forms -inine are right-hand (S)-isomers [4]. The amount and toxin pattern varies between fungal strains, depending on the host plant and geographical region [1,3]. As stated in [6], the degree of variability in the EA pattern in relation to the fungal species and geographical distribution as well as the host plant is not known at present. More data is needed to identify all factors responsible for the variability in the EA pattern in individual plant species [6]. Currently, the regulations are based on the quantities of ergot sclerotia. In Directive 2002/32/EC on undesirable substances in animal feed [7] and its amendment [8], the maximum content of rye ergot (*Claviceps purpurea*) in feed containing unground cereals has been set at 1000 mg/kg. 

However, the physical determination of the contamination rate of cereals by rye ergot is often inaccurate, as the size and mass of the sclerotia may vary considerably, and it is impossible in processed feed and food. Chemical analysis has been suggested for the possible control of potentially contaminated feed and food [6], as various chromatographic methods are available for detecting EAs in feed and food [9,10]. EU Member States were recommended for monitoring data on the presence of EAs in cereals and cereal products and to report their findings to the EFSA [6]. Further, simultaneous determination of the sclerotia content in the samples was recommended to improve the knowledge of the relationship between the content of sclerotia and the level of individual EAs [6].

There are several reports on the presence of EAs in feed from different countries. Ruhland and Tischler [11] reported the occurrence of EAs in 86–100% of feed samples from Germany. The median concentrations in different feeds were 25–96 µg/kg and the maximum concentrations were 149–4883 µg/kg. In a Dutch survey of 184 samples of cereals and compound feed [12], 43% of the cereal samples and 83% of the compound feeds contained EAs with an average concentration of 89 µg/kg and a maximum concentration of 1231 µg/kg. The major detected EAs were Es, Et, Ecr and Ekr. Malysheva et al. [13] presented the occurrence of EAs over a three-year period in a total of 1065 cereal samples originating from 13 European countries. EAs were present in 52% of rye, 27% of wheat and 44% of triticale samples at total EA levels ranging from ≤ 1 µg/kg (≤ LOQ) to 12,340 µg/kg. The most frequently occurring EAs were Es, Ekr and Ecr. An occurrence study in different cereals of the 2012–2014 harvesting season in France was reported by Orlando et al. [14]. The rye and triticale were the most prone to infection. EAs were present in 78% of rye and 54% of triticale samples. In 74% of the samples the most frequent EAs were Et, Es and Ecr. Topi et al. [15] reported EA contamination in Albanian wheat, including 71 samples in the years 2014–2015. In 2014 and 2015, 48.6% and 19.4% of the samples contained EAs, respectively. The EA concentrations were from 10.3 to 975.4 µg/kg. Ecr, Es and Em were the predominant EAs in most samples. The differences in ergot patterns and total EA concentrations are apparently due to the geographical region and environmental conditions during the development of the ergot sclerotia, as mentioned by Krska and Crews in their study [3]. 

Until now, no data on EA in feed in Slovenia were available. For this reason, a visual and chemical method using LC-MS/MS was introduced to determine ergot and EAs (Em, Et, Es, Ecr, Ekr, Ekr and Eco and their –inine epimers) in cereal grains collected in Slovenia in 2014–2017 and for a correlation study between sclerotia content and EA concentration. The approach of combining visual and chemical methods in the correlation studies will contribute to the knowledge of the relationship between the presence of sclerotia and the content of EAs and the EA pattern in different cereal grains. The four-year study focused on cereals to be used as feed material in Slovenia.

## 2. Results

### 2.1. Occurrence of Total Ergot Alkaloids 

A total of 517 ground cereal samples were tested in the four-year study of the presence of EAs in various cereals. EAs were determined in 206 wheat, 136 barley, 101 triticale, 35 rye, 23 spelt and 16 oat samples. The samples containing one or more individual EAs at concentrations equal to or above the LOQ (10 µg/kg) were considered positive. The overall occurrence of EAs within each cereal group in the years 2014–2017 is shown in Table 1. Contamination with at least one of the 12 analysed EAs was revealed in 87 of the 517 samples, which amounts to 17% positive rate. The incidence of positive samples was the highest in rye (54%) and oat (50%) samples. In the other cereals, EAs were detected in lower percentages. The total EA concentrations ranged from 14 to 4217 μg/kg. The overall mean concentration level of the positive samples was 448 μg/kg and the median level was 154 μg/kg. The -ine and -inine epimers were present in 95% and 91% of the contaminated samples, respectively. The maximum concentration levels of the –ine and –inine epimers were 2476 µg/kg (wheat, 2014) and 1849 µg/kg (rye, 2017), respectively. 

The susceptibility of cereals to ergot infection (from most to least) was ranked rye, wheat, triticale, barley, and oats. Rye, as an open pollinator, allows easy access of the fungus into the flowering head, therefore, it is more susceptible to ergot infection than wheat and barley, which are self-pollinators. Oat is rarely affected [9]. However, in our study, a high incidence rate was revealed in oat with a high median concentration, but the maximum concentration of the total EAs was higher in cereals with a higher susceptibility to ergot infection (rye and wheat). The maximum concentration levels of total EAs followed the order wheat (4217 µg/kg, 2014), rye (4114 µg/kg, 2017), spelt (2682 µg/kg, 2014), triticale (2587 µg/kg, 2014), oat (2191 µg/kg, 2016), and barley (1177 µg/kg, 2016). 

**Figure 2:**
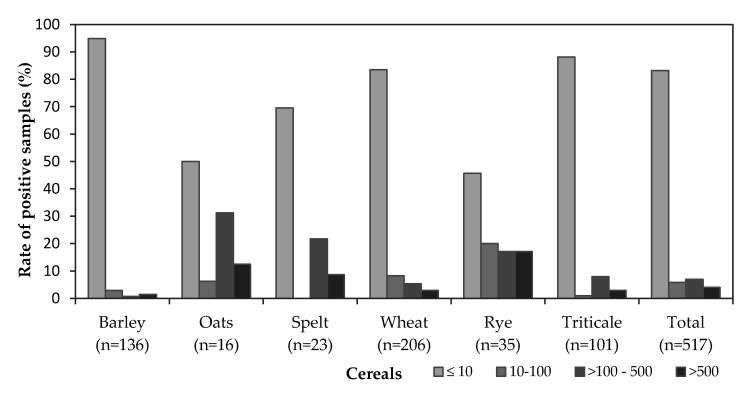
Distribution of the total ergot alkaloid concentrations (µg/kg) in the samples analysed in the years 2014–2017. *n*: number of samples.

The distribution according to total EA concentrations in the analysed samples is shown in Figure 2. Within each cereal group, more than 50% of the results were below the LOQ (10 µg/kg). Of the 517 samples analysed, in 430 samples (83%) the concentrations of total EAs were less than the LOQ. Only 17% of the samples contained EAs with concentrations between 14 µg/kg and 4217 µg/kg. The highest percent of samples with an EA concentration below the LOQ was observed in barley (95%), followed by triticale (88%), wheat (84%), spelt (70%) and oat (50%). Of the 35 rye samples analysed, 16 (46%) contained no EA, while the other 19 samples contained very equally distributed EA concentrations between the LOQ and the maximum (4114 µg/kg, Figure 2). The mean concentration of 502 µg/kg was not substantially different from the mean concentration obtained in oat (594 µg/kg) (Table 1). Of the eight contaminated oat samples, five (31%) had an EA concentration between 100 and 500 µg/kg and two had an EA concentration of more than 500 µg/kg, which contributed to a higher mean concentration level in oat samples. Similarly, in all contaminated spelt samples, the concentrations were between 100 µg/kg and 2682 µg/kg, while no samples with concentrations between the LOQ and 100 µg/kg were found. Therefore, the mean concentration was higher in spelt samples than in rye samples (715 µg/kg). 

**Figure 3:**
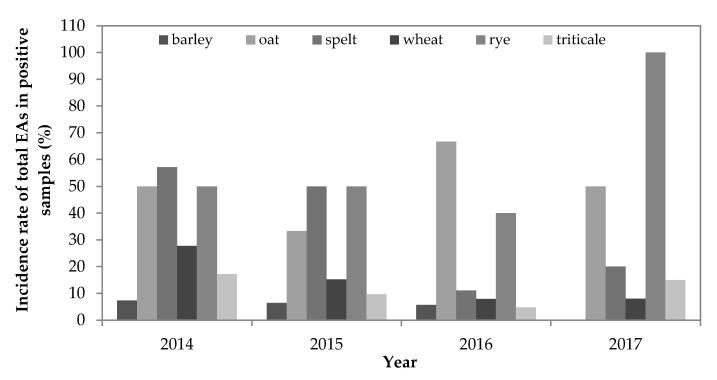
Yearly incidence of total ergot alkaloids (%) in the analysed samples. The samples containing one or more individual EAs at concentrations equal to or above the LOQ (10 µg/kg) were considered positive.

The yearly occurrence of total EAs is shown in Figure 3. In the years 2014–2017, EAs were found in 25%, 15%, 10% and 13% of the tested samples, respectively. In 2014, the highest EA incidence was observed in oat, spelt and rye samples, where at least 50% of the samples of each cereal contained one or more EA. The incidences of EAs in spelt and rye samples were similar in 2014 and 2015, while that of oat was lower (33%) in 2015. In 2014, an increased incidence in wheat and triticale was also observed compared to 2015–2017. In 2016 and 2017, the highest incidence of EAs was observed in oat and rye samples, where more than 40% of the samples were contaminated with one or more EA, while contamination of other cereal samples was less than 20%. In 2017, all rye samples contained EAs. The incidence of EAs in spelt, wheat and triticale was the lowest in 2016, with an increased incidence in spelt and triticale in 2017. A review of the observed years indicated that the incidence of EAs in the analysed samples was the highest in 2014, and it decreased in the subsequent years, except for those of rye and oat. 

In each cereal group, the differences according to all observed years were also evaluated. The *p*-values for barley, oat, spelt, rye and triticale were 0.564, 0.880, 0.255, 0.492 and 0.543, respectively. In the mentioned cereal groups, we have not found significant differences in different years; only in the wheat cereal group (*p* = 0.005) was a significant difference observed. 

**Figure 4:**
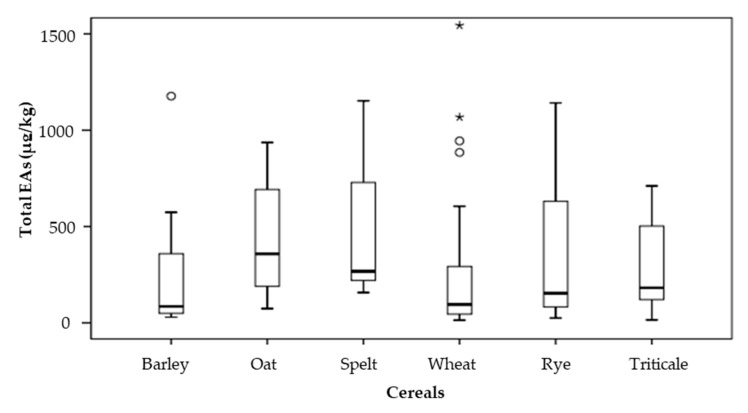
Box-plot graph summarizing the total content of 12 ergot alkaloids (as shown in the legend of Figure 7) in the positive samples (containing at least one EA with a concentration of 10 µg/kg or more) for each cereal species in the years 2014–2017. Median concentrations are indicated by horizontal lines in the boxes encompassing the 25th–75th percentiles. Outliers in the 95th percentiles are indicated as empty dots (○) and stars (*).

The total EA concentrations in the years 2014–2017 are shown in Figure 4. Since some samples had a very high total EA concentration, the mean concentrations of all cereals are higher than the median concentrations. Due to the higher ratio of results with a concentration of EAs above 500 µg/kg, the median concentration in oat and spelt samples was higher than that of the rye samples. The median concentration, from highest to lowest, was: oat (359 µg/kg), spelt (264 µg/kg), rye (154 µg/kg), triticale (152 µg/kg), barley (111 µg/kg) and wheat (102 µg/kg).

The differences between the mean values of total EA concentrations among the years (2014–2017) were evaluated using the Kruskal–Wallis test. The differences were considered significant if *p* < 0.05. The mean values of total EAs concentrations were significantly different (*p* < 0.001) between the years 2014–2017. Furthermore, the mean EA concentrations in the year 2014 were higher than in 2015, 2016 and 2017 (*p* < 0.001), in the year 2015 they were lower than in 2016 (*p* = 0.123) and 2017 (*p* = 0.507) and in the year 2016 they were lower than 2017 (*p* = 0.452).

Statistical differences with *p* < 0.05 in the mean values of total EA concentrations were found between different cereal groups. The total mean EA concentration in barley was higher than in all other cereal groups (oat, spelt, wheat, rye and triticale) with *p* < 0.001. The total mean EA concentration in spelt was higher than in triticale (*p* = 0.047) and barley (*p* < 0.001), but lower than in the other cereals (*p*-values between 0.082 and 0.317). The total mean EA concentrations were higher in oat than in wheat, barley and triticale (*p* < 0.001), while it was lower in oat than in spelt (*p* = 0.167) and rye (*p* = 0.582). No significant difference was found between wheat and triticale (*p* = 0.478), but the total mean EA concentration in wheat was higher than in barley, oat and rye (*p* < 0.001). In addition, the total mean EA concentration in rye was higher than in triticale, wheat and barley (*p* < 0.001). 

### 2.2. Co-Occurrence of Ergot Alkaloids 

**Figure 5:**
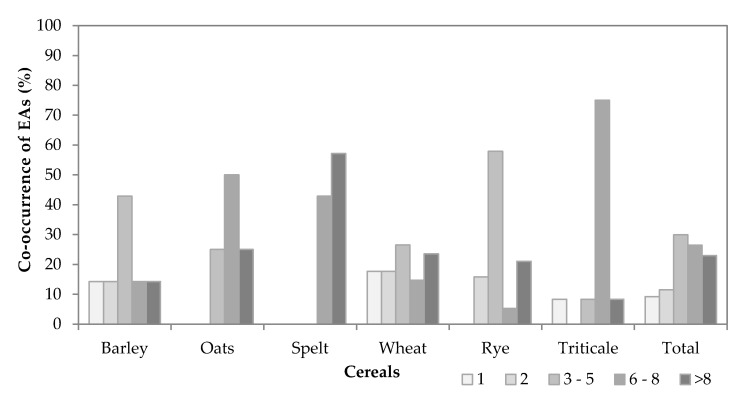
Co-occurrence of EAs (as indicated in the legend of Figure 7). Only positive samples are considered (containing at least one EA with a concentration of 10 µg/kg or more).

The co-occurrence of EAs in positive samples of each cereal and in all positive samples together is shown in Figure 5. Of the 87 positive samples, eight (9.2%) and 10 (11.5%) samples contained only one or two EAs, respectively. Three or more and six or more EAs were present in 79.3% and 49.4% of all samples, respectively.

A difference in the co-occurrence of EAs was found between oat, spelt and triticale on one side and barley, wheat and rye on the other side. In oat, spelt and triticale, the positive samples were contaminated with at least three EAs, but in 75–100% of the samples six or more EAs were present. In barley, wheat and rye, more than 50% of the positive samples contained fewer than six EAs. In the spelt samples, all positive samples contained six or more EAs per sample; in oat and triticale samples, the co-occurrence exceeded 75%; while in barley, rye and wheat, it was lower than 40% (Figure 5). 

In spelt, 43% of the positive samples contained six to eight co-occurring EAs, while 57% contained more than eight EAs. Six to eight or more EAs were also found in oat (50%) and triticale (75%) samples, whereas the co-occurrence of more than eight EAs was found in 25% or fewer certain cereal samples. The co-occurrence of three to five EAs was the highest in rye (58%) and barley (43%), followed by wheat (27%) and oat (25%), while the lowest co-occurrence was observed in triticale (8%). An equal distribution of co-occurrences over a complete range from one to more than eight EAs was observed in wheat samples. The co-occurrence of three to five EAs and more than eight EAs was 27% and 24%, respectively, while the other co-occurrences were approximately 15%. 

**Figure 6:**
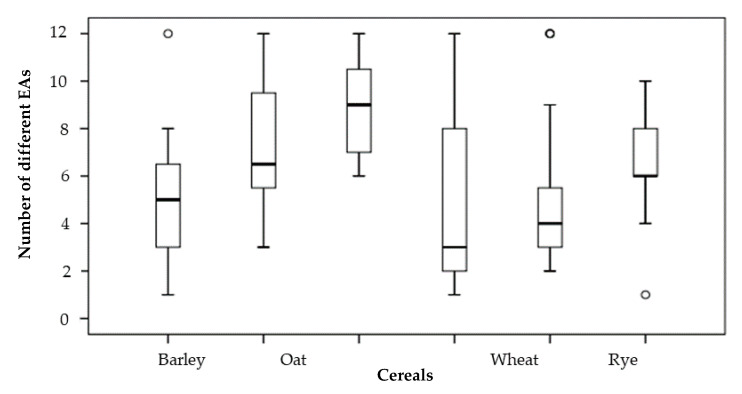
Number of different ergot alkaloids in cereals. Mean values are indicated by horizontal lines in the boxes encompassing the 25th–75th percentiles. Outliers in the 95th percentiles are indicated as empty dots (○).

A statistically significant difference (*p* < 0.05) between the mean value of the number of co-occurring EAs in positive samples and the type of cereal (*p* = 0.035) was found. The results are presented in Figure 6. The mean of EA number in spelt samples was greater than in barley (*p* = 0.046), triticale (*p* = 0.043), rye (*p* = 0.010) and wheat samples (*p* = 0.019). All other co-occurrences of EAs in cereals were not significantly different (*p* = 0.053–0.812).

### 2.3. Individual Ergot Alkaloids

**Figure 7:**
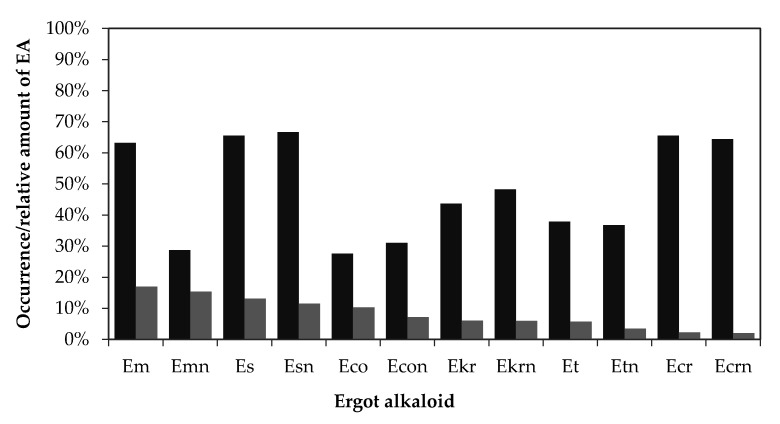
Occurrences (dark bars, % of 87 positive samples) and relative amounts (light bars, % of the sum of individual EA concentration compared to the sum of total EA concentration (39100 µg/kg)) of the individual EAs in the positive cereal samples. Legend: Em—ergometrine, Emn—ergometrinine, Es—ergosine, Esn—ergosinine, Eco—ergocornine, Econ—ergocorninine, Ekr—ergocryptine, Ekrn—ergocryptinine, Et—ergotamine, Etn—ergotaminine, Ecr—ergocristine and Ecrn—ergocristinine.

In 87 positive samples, all EA forms were found, but some EA forms were observed more often than others (Figure 7, dark bars). The occurrence of individual EAs in the positive samples was evaluated as the ratio (%) between the number of an individual EA and the number of the positive samples. The most frequently occurring alkaloids in the positive samples were Em, Es, ergosinine (Esn), Ecr and ergocristinine (Ecrn). Es and Esn were present in 57 (66%) and 58 (67%) of the samples, respectively, while Ecr and Ecrn were found in 57 (66%) and 56 (64%) of the 87 positive samples. Em had a higher rate of occurrence (63%) than the corresponding epimer Emn, which was found in only 29% of the positive samples. 

The relative EA amounts (%) of individual EAs present in the positive samples are shown in Figure 7 with light bars. The relative amounts were calculated as the ratio of the sum of the individual EA concentration to the sum of the total EA concentration in the positive samples. The relative amounts of -ine epimers were higher than those of -inine epimers. The predominant EA was Em (17%) and its epimer Emn (15.3%), followed by Es (13%), Esn (11.5%), Eco (10.4%) and Econ (7.2%). The most abundant EAs were the Em/Emn and Es/Esn epimers.

**Figure 8:**
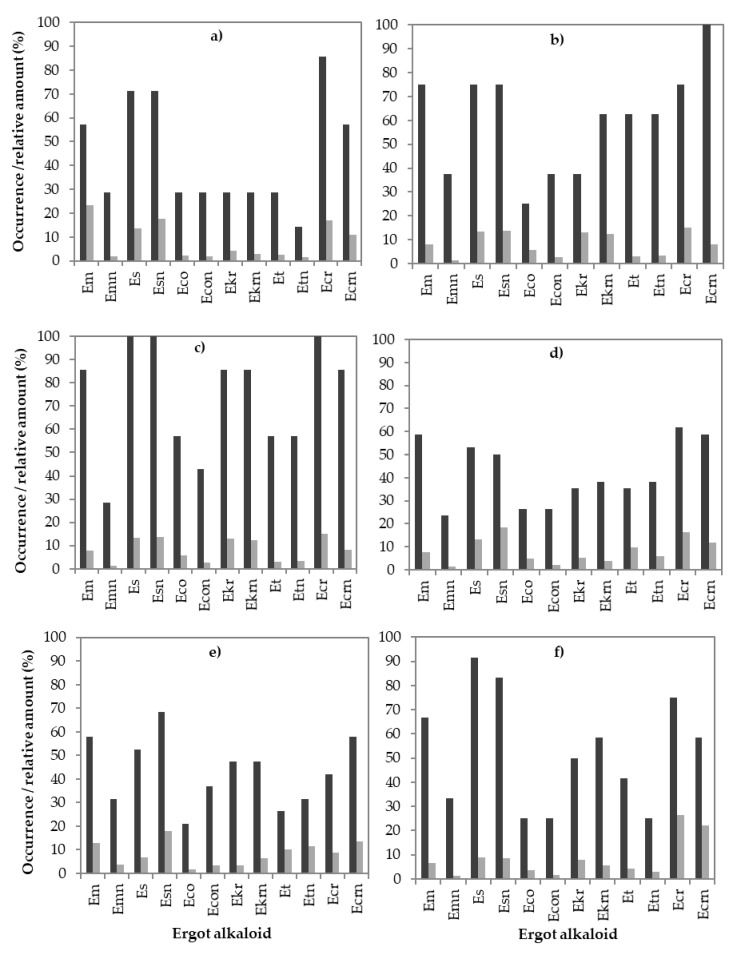
Occurrences (dark bars, % of 87 positive samples) and relative amounts (light bars, % of the sum of individual EA concentration compared to the sum of total EA concentration (39,100 µg/kg)) of the individual EAs in different cereals: (**a**) barley, (**b**) oat, (**c**) spelt, (**d**) wheat, (**e**) rye and (**f**) triticale. Only positive samples are considered (containing at least one EA with a concentration of 10 µg/kg or more).

The occurrence of individual EAs in the positive samples varied depending on the cereal group (Figure 8). All twelve individual EAs were found in all observed cereal groups, but with different EA patterns and different relative EA amounts. Em, Es, Esn and Ecr, Ecrn were determined in 58–100% of all positive samples for a specific cereal. Em was most frequently found in spelt (86%), oat (75%) and triticale (67%), while in barley, wheat, and rye it was slightly lower (58%). Emn was present in 24–38% of the positive samples in all cereals, with the highest occurrence in oat and the lowest in wheat. Es and Esn were present in spelt in all positive samples (100%) and in 92% and 83% of the positive triticale samples, respectively. The epimers Es and Esn were present in 75% of oat and 71% of barley but in less than 70% of wheat and rye. More barley, spelt, wheat and triticale samples were contaminated by Ecr than by its epimer, Ecrn, which was higher in occurrence in oat and rye. Eco, Ekr, Et and their corresponding epimers (Econ, Ekrn and Etn) were infrequently present in the positive samples. Et and Etn were found in more than 50% of the positive oat and spelt samples, Ekr and Ekrn were found in oat, spelt and triticale samples, while more often Eco was found only in spelt. The occurrence of Et ranged from 26% in rye to 62.5% in oat, while the Etn level ranged from 14% in barley to 62.5% in oat. Ekr and Ekrn were present in 86% of the positive samples of spelt, while their lowest level was observed in barley (28.5%). 

The relative amounts of individual EAs differed between cereal groups. In all samples, both epimeric forms, –ine and –inine, were found, however, they did not always appear together. In most cases, –ine forms were present in a higher ratio than their epimers (–inine form) with one exception (the epimers Es and Esn). In all cereals, Esn rather than Es was the predominant alkaloid at a ratio of 8.5–25%. If a higher occurrence of an individual EA was found in a positive sample, usually a higher ratio was observed. 

### 2.4. Ergot (Sclerotia) Content and Analytical Correlation Study

Of the 432 samples analysed in the years 2014–2016, the EAs were found in 76 samples. However, with microscopic inspection, sclerotia were found in only 52 positive samples. In the 76 samples, the mean and maximum concentrations of the total EAs were 439 µg/kg and 4217 µg/kg, respectively. The mean and median sclerotia weights in the samples were 134 mg and 36.5 mg, respectively. The average sclerotia content per kg of cereal was 0.14 g/kg. The most abundant individual EAs in the samples were Em, Es, Esn, Ecr and Ecrn (58–67%), followed by Ekr, Ekrn, Et and Etn (40–49%). The contributions of Eco, Econ and Emn were 29% or less.

A Spearman’s rank-order correlation was run to determine the relationship between the mass of sclerotia (mg) or sclerotia content (g/kg) and the total EAs concentrations (µg/kg) as well as individual EA concentrations (Table S1). The results were evaluated as statistically significant at the level *p* < 0.01 or 0.05 and the strength of the correlation was determined by comparing calculated correlation coefficient values (r) with Evans guidelines (Table S2) [16]. There was a weak, positive, statistically significant correlation between the mass of sclerotia or sclerotia content and the total EA concentrations (r = 0.284, *p* = 0.008; r = 0.315, *p* = 0.003). Furthermore, the concentrations of all individual EAs were significantly different with *p* < 0.01 or 0.05 and were weakly or moderately correlated according to the correlation coefficient values shown in Table S2. There was a strong, positive, statistically significant (r ≥ 0.65, *p* < 0.001) correlation between each EA pair –ine and –inine.

In a further experiment, a visual screening before chemical analysis of cereal samples collected in the year 2017 was performed. A total of 85 cereal samples were analysed. The results for the visual inspection as well as the chemical determination are shown in Table 2. In 65 samples (76.4%, denoted A and D in Table 2), the results of the visual inspection for the presence and absence of sclerotia, matches with the result of the chemical analysis. In 20 of the positive samples (denoted B), sclerotia were found with an absence of EAs. Cases where no sclerotia were found although alkaloids appeared to be present were none. The average total EA concentration in cereals containing sclerotia was 512 µg/kg and the median concentration was 139 µg/kg. In 11 samples, where the masses of sclerotia and the EAs were determined, the most frequently present individual EAs were Es, Ecr and Em. Compared to the present study, much higher average levels were found in cereals in the study by Mulder et al. [12].

## 3. Discussion

### 3.1. Occurrence of Total Ergot Alkaloids

In this study, 517 samples of cereals were analysed for EA content. There are only a few available surveys on the occurrence of EAs focusing on feed. In Table 3, an overview of the results from different studies is presented. Considering all samples in a specific cereal group, the incidence of positive samples in Slovenian cereals was the highest in rye (54%), followed by oat and spelt (50% and 30%), while wheat and triticale were lower (17% and 13%). Remarkably, the incidence of positive samples in rye was almost identical to the values reported in Europe (52%) and the Netherlands (50%) [12,13] but differed from the results of Ruhland and Tischler [11], who observed EA contamination in all 15 analysed rye samples and also a high incidence rate for other grains from Germany with levels from 86% to 93%. The incidence of EAs in wheat samples in our study was much lower than those in studies from Germany, the Netherlands and the USA [11,12,17], but close to the results from Europe [13]. In the study, more than half of the wheat samples originating from Germany, Poland, Switzerland and the Czech Republic did not contain EAs, which is also similar to our results. Out of 206 Slovenian wheat samples, 83% were not contaminated with EAs. The incidence of EAs in triticale samples was similar to those reported in the studies of Malysheva et al. [13] and Mulder et al. [12], where 44% and 33.3% of 27 and 45 analysed samples, respectively, were contaminated. The limited number of samples in the European study did not allow a robust analysis as conducted by Malysheva et al. [13] and could also be the reason for a slightly higher incidence rate of positive samples in European samples than in Slovenian samples, where results were obtained from 101 analysed samples. The highest incidence rate in triticale samples was observed in 14 German samples (93%) and the lowest was observed in our samples (13%), as shown in Table 3. The literature results for the incidence of EAs in barley are scarce. Tittlemier et al. [18] analysed a total of 25 barley samples, but the incidence rate was not provided. In our study, most of the 136 analysed barley samples were uncontaminated. In 4% of the positive samples, the maximum and median concentrations were 1177 µg/kg and 111 µg/kg, respectively.

Topi et al. [15] reported on the presence of EAs in 71 wheat samples collected during the years 2014 and 2015. The maximum concentration of EAs was 975.4 µg/kg, which was lower than our result (4217 µg/kg). A similar maximum content of total EAs was observed for American wheat samples (4760 µg/kg), while in other countries (Canada, Europe, Germany, the Netherlands) the maximum concentrations in wheat samples were from 529 µg/kg to 1236 µg/kg (Table 3), but median concentrations were from <1 ug/kg (< LOQ) to 195 µg/kg. The highest median concentration level was observed in Albanian wheat (226.7 µg/kg). The median concentration in Slovenian wheat samples was between the values obtained in the samples collected in European countries (< LOQ) and in Albania (226.7 µg/kg). Of the 35 rye samples analysed, the median and maximum concentrations of 154 µg/kg and 4114 µg/kg were determined, respectively. A similar maximum concentration (4850 µg/kg) was reported by Meister and Batt [19]. Malysheva and co-workers [13] reported a much higher maximum concentration (12,340 µg/kg), which was found in a rye sample from Switzerland, while the results for the samples from the other countries mentioned in their work were below 500 µg/kg. Other studies performed by Mulder et al. [12] and Ruhland and Tischler [11], demonstrated lower maximum concentrations. In Dutch and German rye, it was 1231 µg/kg and 1067 µg/kg, respectively. The higher concentration in rye samples was expected because it is particularly susceptible to infection by *C. purpurea*, as rye is a cross-pollinator with opened florets [9].

In the German study [11] and triticale samples from different countries of Europe [13], the maximum concentration was the same (1103 µg/kg), with a median concentration of 25 µg/kg and < LOQ. In the European study, in 73% of all analysed samples, the results were below the LOQ. The statistical approach of Malysheva and co-workers [13] included not only positive samples but also the results of all samples. The median concentration in their study was below the LOQ for wheat and triticale samples and equalled the LOQ (1 µg/kg) for rye samples. The concentration considering only positive samples was 152 µg/kg. This result was higher than those in positive samples from the Netherlands (63.9 µg/kg) and Germany (25 µg/kg), where the number of analysed samples was much lower. If the same approach as that of Malysheva et al. [13] had been used in our study, the median concentration would also have been < LOQ, because in our case the ratio of the results below the LOQ was very high (83%). Additionally, in our study, we also presented the occurrence of total EAs in spelt and oat. There are no data in the literature to compare to these results. The maximum levels of total EAs in oat and spelt samples were similar (2191 µg/kg and 2682 µg/kg, respectively) as in triticale samples (2587 µg/kg). The median concentration was higher in oat samples, as in spelt samples. Since the number of samples was low, the data represent a rough evaluation of the EA occurrence in the two cereals in the years 2014–2017. 

### 3.2. Co-Occurrence and Individual Ergot Alkaloids

In the contaminated samples of the four-year survey, all EAs were found. In the analysed samples, the number of co-occurring EAs was usually higher than three EAs. The results are shown in Figure 6. Only one EA was present in 9% of all samples, with the highest occurrence in barley, wheat and triticale samples. However, in all oat, spelt and rye samples, more than one EA was found. The co-occurrence of two EAs was revealed in barley, wheat, rye samples, but not in triticale samples. Three to five EAs co-occurred mostly in rye (58%) and barley (43%) samples but were not found in spelt samples, which usually contained six or even more than eight EAs. Similar high co-occurrences of more than six EAs were observed in triticale samples, with a lower co-occurrence of more than eight EAs. A difference in the co-occurrences of EAs was found between oat, spelt and triticale positive samples and between barley, wheat and rye positive samples. In oat, spelt and triticale, the positive samples were contaminated with at least three EAs, but in 75–100% of the samples, six or more EAs were present. Furthermore, no EA can be used to predict the total EA concentrations, because none of the EAs were determined in all contaminated samples in all cereals.

In most analysed samples, the –ine and –inine forms of EAs were found together. The occurrence of a single EA was observed for Emn and Et in barley. The distribution of EAs in positive samples is evident in Figure 7 and Figure 8. The predominant EAs were Es and Ecr with the corresponding epimers and Em. The less frequently occurring EAs were Emn, Eco, and Et with the corresponding epimers, as they were present in 21–42% of the positive samples. However, the least frequently occurring EAs were Eco and the corresponding epimers Econ and Emn, which were found in less than 30% of the positive samples. A scarce amount of data is available on the EA pattern in grains or feed. Topi et al. [15] reported the ergot pattern for wheat samples harvested from 2014 to 2015. In the year 2014, the most frequently occurring EAs were Em and Es, which were present in 76.5% and 70.6% of the positives, respectively. The least frequently occurring EAs were Econ and Ekrn in 5.9% and 11.8% of the positive samples, respectively. The predominant EAs in the Slovenian wheat samples were Ecr and its epimer Ecrn, which were present in 61.8% and 58.8% of the positives, respectively. In our wheat samples, Es and Em were also found, as in Albanian wheat, but less frequently. They were present in 58.8% and 52.9% of the positives, respectively. In the Albanian samples from the year 2015 [15], the predominant EA was Ecr, which was present in 71.4% of the positive samples. Ecr and Ecrn were also the predominant alkaloids in wheat samples in Canada and France, followed by Et [14,18]. In contrast, in wheat samples from Belgium, only Em and Emn were detected, while the study of European samples concluded that Es, Ecr, and Ekr were the predominant EAs in most samples [13]. In our study, in more than 70% of the barley samples, the predominant EAs were Es and Ecr and their epimers, which were observed also in oat, spelt and triticale samples and were slightly less predominant in wheat and rye. Additionally, Em was present in more than 57% of all cereal positive samples, while Emn was the predominant EA in oat, rye, and triticale, but in other cereals its level was below 30%. Ekr and Ekrn were usually found in less than 40% of the positive samples for a specific cereal, with the highest occurrence in spelt samples. The least frequently present EAs in barley positive samples were Eco, Et and Ekr and the corresponding epimers, which were present in less than 30% of the positives. A similar low occurrence of Eco, Et, and Ekr was observed in wheat. In rye and triticale, only the Eco and Et occurrences were less than 40%, but Ekr was higher (47.4–58.3%). Overall, we agree with the conclusion of Malysheva et al. [13] and Orlando et al. [14] that the EA patterns and alkaloid levels found in the samples depend on the cereal type. For the cereals we analysed, such as barley, oat, spelt, and triticale, no literature data is available for a results comparison.

The geographical region and environmental conditions, the weather condition shortly before and during the flowering period of the cereal, and data on cereal cultivars and *Claviceps* species infecting the individual sample should be taken into account [3,20] as reasons for the differences in the EA pattern and EA contamination. These data were not collected in the study and, therefore, no conclusions can be drawn about the correlation of EA pattern and EA contamination on geographical and climatic conditions or *Claviceps* species. However, the available data from the meteorological stations show that the annual average temperatures in all years (2014–2017) were above the long-term average (1981–2010), in 2014 even by more than 1.6 °C. The fluctuations in annual average temperatures depend on both the year and the region. Annual precipitation in the period 2014–2017 fluctuated significantly compared to the long-term average (1981–2010), both by year and by individual regions of Slovenia. In 2014 there was significantly more precipitation (from 5% to more than 40% more) than the long-term average (1981–2010). In 2016 and 2017, some regions also received more precipitation than the long-term average, but the deviation was slightly smaller (0–20%). 2015 was drier than the long-term average in most regions.

### 3.3. Sclerotia Mass and Analytical Correlation Study

The visual screening method is a great supporting warning tool for ergot contamination, but only in combination with a chemical method for determination of EAs because, in some cases, the ergot bodies are present in samples as dust and with the visual method a false negative result could be produced, which is well apparent from our results in Table 2. With a chemical analysis, we also determined the presence of EAs in the samples where the results for visual screening were negative. 

The objective of this study was to determine correlations between the EA concentrations of the individual EA and the total concentrations of EA as well as between the sclerotia contents and the EA concentrations. The results are shown in Table S1. Most EAs correlated with each other, but with no specific predominant individual EA, which could be used as a marker for determining the total EA concentration based on the content of a specific single EA in the sample. The results were in accord with the information available in the literature [12,21]. In contrast, a strong linear relationship between the concentration of ergot alkaloids and the presence of ergot sclerotia was reported by Tittlemier et al. [18] in Canadian wheat and other cereals and by Orlando et al. [14] in French cereals. 

The results indicate that a higher sclerotia mass does not necessarily mean a higher total EA concentration in the sample. A fluctuation of the total EA concentrations in the samples with sclerotia must be expected because of the variability of the sclerotia mass and size in the samples. Furthermore, the fluctuation of the total EA concentration also varies between samples with equal sclerotia masses. The concentration of ergot body in cereals is for an animal restricted to 1000 mg/kg in feed materials and compound feed containing unground cereals [7,8], but the sclerotia content in unground cereals could not be used as an indicator of the EA contamination level of cereals intended as animal feed, because of the fluctuation of total EA concentration. Both methods, visual and chemical, should be used to monitor the EA in feed as recommended in [6]. Because of the diversity of EA patterns in cereals, the total EA concentration could be used to set the maximum level of EAs in cereals intended for animal feeding.

## 4. Conclusions

In this work, the occurrence of EAs in cereals in Slovenia was presented for the first time. The study contributed to the data on the EA content in feed material in Southeastern Europe, which are still scarce. The combination of visual and chemical methods improved the knowledge about the relationship between the content of sclerotia and the level of individual EA. The presented data outlined the importance of establishing maximal permitted levels of EA in feed and food. The maximum permitted level based on the presence of sclerotia [7,8] is not sufficient because a weak correlation between content of ergot sclerotia and the concentrations levels of individual and total EAs was observed. All individual EAs correlated with each other, but a specific EA that could be used as a marker for the determination of EAs in cereals was not found. As several cereal samples are contaminated with EAs, farmers still need to manage ergot to reduce the risk of occurrence of EA by using preventive methods to manage EAs concentrations. It is recommended that the official control of cereals continues with both visual and chemical methods.

## 5. Materials and Methods

### 5.1. Sample Collection

The samples (wheat, barley, triticale, rye, oat, and spelt) were collected in all regions of Slovenia in the years 2014–2017. The samples were taken according to Commission Regulation (EC) No. 152/2009 [22] and its amendment [23]. A total of 517 samples (173 samples in 2014, 123 in 2015, 136 in 2016 and 85 in 2017) were collected and tested for the presence of EAs and for sclerotia using visual and mass analyses. For the preparation of the final samples, the collected samples were divided into equal amounts using a divider according to Commission Regulation (EC) No. 152/2009 [22] and its amendment [23]. 

### 5.2. Standards and Chemicals

The EA standards Em, Es, Et, Eco, Ekr, Ecr and their -inine epimers, as well as MycoSep 150 Ergot columns were purchased from Romer (Biopure, Tulln, Austria). Stock standard solutions and the mixed working standard solutions were prepared in acetonitrile and stored in amber glass vials at −20 °C. The certified purity of individual standard substances was between 95.1% ± 4.9% and 99.0% ± 1.0%. The concentrations of the –ine and –inine stock standard solutions were 100 and 25 μg/mL, respectively. However, the exact concentrations of individual EA stock standard solutions obtained by reconstituting the content of ampoules in 5 mL of acetonitrile were provided by the producer. Acetonitrile (Honeywell, NC, USA) and ammonium carbonate (Merck, Darmstadt, Germany) were of analytical or LC-MS-grade purity. Deionised water was prepared using a Milli-Q system (Millipore, Bedford, MA, USA). The extraction solution was a mixture of acetonitrile and a solution of ammonium carbonate (0.2 g of ammonium carbonate per 1 L of deionised water) in the ratio of 84:16 (*v*/*v*). For the final sample solution, the two components were mixed in the ratio of 50:50 (*v*/*v*). Mobile phase component A was deionised water containing 0.2 g of ammonium carbonate per litre and component B was acetonitrile containing 0.1% of formic acid per litre.

### 5.3. Analytical Procedure

For the determination of EAs, a procedure described by Topi et al. [15] was used. It was based on the procedures of Mulder et al. [12]; Diana Di Mavungu et al. [24]; Kokkonen and Jestoi [25]; Crews et al. [26]; and Krska et al. [27]. Sample preparation included the extraction of EAs from ground cereals (20 g) with 100 mL of extraction solution. After extraction, 4 mL of the extract was transferred to a glass tube and purified by passing it through a Mycosept 150 Ergot column (Romer, Biopure, Tulln, Austria). Afterwards, 1 mL of the extract was evaporated to dryness under a vacuum at + 60 °C (Büchi Labortechnik AG, Flawil, Switzerland) and redissolved in 500 µL of a mixture of acetonitrile and ammonium carbonate in the ratio of 50:50. Chemical analysis of the EAs was performed with an LC-MS/MS. Chromatographic separation was performed with an Acquity UPLC (Waters, MS, USA) on a 2.7 μm Ascentis Express Phenyl-hexyl column, 2.1 × 100 mm (Supelco, Bellefonte, PA, USA). Two components of the mobile phase were mixed in a gradient mode. The starting composition of the eluent was 95% A and 5% B. The portion of component B was linearly increased to 25% within 1 min and further increased to 60% within the next 7 min. In the next 0.1 min, the proportion of component B was returned to 5% and then held for 5 min. The mobile phase flow rate was 100 μL/min and the column temperature was 30 °C. MS/MS analysis was performed using an ESI+ and a triple quadrupole mass spectrometer operated in MRM mode. The capillary voltage was 3.5 kV, the desolvation temperature was 500 °C, and the ion source temperature was 150 °C. The retention times of single EAs and monitored transitions are provided in Table 4. Quantification was performed using matrix-matched calibration. However, no internal standard was used.

### 5.4. Method Validation

The validation was performed using cereals and compound feed samples spiked with each EA at the concentration levels of 10, 200 and 500 μg/kg. The method validation procedure and validation results were presented in our previous work [15]. The recovery rates ranged from 100% to 124%. The intra-day and inter-day precisions of EAs expressed as the relative standard deviation (RSD_r_ and RSD_R_, respectively) were from 5.8% to 17% and from 7% to 35%, respectively. The results are shown in Table S3 in the supplementary section. Essentially, the limit of detection (LOD) and the limit of quantification (LOQ) of single EAs were estimated as concentrations resulting in a signal-to-noise ratio of 3:1 and 10:1, respectively. However, as the limit of quantification (LOQ) of single EAs, the lowest tested concentration of 10 µg/kg was accepted according to the needs of the clients. 

### 5.5. Ergot (Sclerotia) Content and Analytical Correlation Study

In the years 2014–2016, the collected samples were divided into two reduced samples using a divider. One reduced sample was used for chemical analysis of EAs and the second for sclerotia visual analysis and sclerotia mass determination. The chemical determination of EAs was conducted with an LC-MS/MS as described above. Sclerotia were visually identified in each sample by their purplish/black colour and their cylindrical shape with round ends using the microscopic method recommended by the International Association of Feedingstaff Analysis (IAG) [28]. Sclerotia were removed from a sample and weighed, and the ergot concentration was calculated. The sclerotia mass (mg) was determined only in cereal samples contaminated by EAs (*n* = 76). 

In the year 2017, all samples were visually inspected for sclerotia before chemical analysis. Sclerotia were removed from a sample, visually identified as mentioned above and weighed, and the ergot concentration was calculated. Afterwards, sclerotia were returned to the original sample. It was homogenized and ground for the determination of EAs.

### 5.6. Statistical Evaluation

The statistical analysis was performed using SPSS Statistics 23 (IBM, Armonk, NY, USA) [29]. All calculations were performed using a nonparametric approach because of the small samples. The differences in the occurrence of EAs among the years as well as among the different types of cereals were tested using the Kruskal–Wallis test and Mann–Whitney test using the medium-bound approach for left-censored results. The correlations between the mass or ergot concentration in cereals and occurrences of individual or total EAs in positive samples were calculated using the Spearman’s correlation coefficient. For all tests, *p* < 0.05 was considered statistically significant.

## Figures and Tables

**Table 1:** Ergot alkaloid occurrence in cereals in the years 2014–2017.

Descriptive Statistics	Wheat	Barley	Triticale	Rye	Spelt	Oat	Total-ines	Total -inines	Total EAs
Number of samples	206	136	101	35	23	16	517	517	517
Number of positive samples	34	6	13	19	7	8	83	79	87
Incidence of positive samples (%)	17	4	13	54	30	50	16	15	17
Min (µg/kg)	14	27	14	25	152	84	10	9.9	14
Max (µg/kg)	4217	1177	2587	4114	2682	2191	2476	1849	4217
Mean (µg/kg)	363	340	417	502	715	594	252	229	448
Median (µg/kg)	102	111	152	154	264	359	84	82	154

**Table 2:** The number of the positive and negative samples for the visual screening and for the chemical analysis. *n*: number of samples.

Combined Results (*n* = 85)	Sample Group	*n*	%
Visually positive/chemically positive	A	11	12.9
Visually positive/chemically negative	B	20	23.5
Visually negative/chemically positive	C	0	0.0
Visually negative/chemically negative	D	54	63.5

**Table 3:** The occurrence of ergot alkaloids (total) in the cereals. Summary of available data.

Country	Year of Sampling	Feed	Number of Samples	Positive Sample Rate (%)	Median (µg/kg)	Max (µg/kg)	LOD/LOQ (µg/kg)	Method of Analysis	Reference
Slovenia	2014–2017	Wheat Barley Triticale Rye Spelt Oat	206 136 101 35 23 16	17 4 13 54 30 50	102 * 111 * 152 * 154 * 264 * 359 *	4217 1177 2587 4114 2682 2191	3/10	LC-MS/MS	This study
Albania	2014–2015	Wheat	71	33.8	226.7 *	975.4	3/10	LC-MS/MS	[15]
Germany	2013	Rye	60	67	237 *	4850	–	LC-MS/MS	[19]
Canada	2010–2012	Wheat Barley Rye	117 25 1	– – –	195 47 –	666 584 149	-/2	LC-MS/MS	[18]
Europe	2009–2012	Rye Wheat Triticale	157 137 27	52 27 44	1 <LOQ <LOQ	12,340 701 1103	-/1	LC-MS/MS	[13]
USA	2011	Wheat	10	70	85 *	4760	–	LC-MS/MS	[17]
The Netherlands	2007–2010	Rye Triticale Wheat Other cereals	69 45 18 4	50.7 33.3 38.9 25.0	121 * 63.9 * 56 * 961 *	1231 297 529 961	2/10	LC-MS/MS	[12]
Germany	2005–2007	Rye Triticale Wheat Other grains	15 14 21 14	100 93 86 93	96 * 25 * 29 * 44 *	1067 1103 1236 140	5/10	HPLC-FLD	[11]

* Notes: only positive samples considered.

**Table 4:** Retention times of ergot alkaloids and monitored transitions.

Ergot Alkaloid	Retention Time (min)	Precursor Ion (*m*/*z*)	Product Ion 1 (*m*/*z*)	Product Ion 2 (*m*/*z*)
Ergometrine	4.38	326.22	223.17	208.07
Ergometrinine	4.74	326.20	180.18	223.16
Ergosine	6.56	548.35	208.06	268.15
Ergosinine	6.75	548.41	223.16	277.19
Ergocornine	7.08	562.35	208.05	268.20
Ergocorninine	7.51	562.35	223.09	277.18
Ergocryptine	7.47	576.35	223.09	208.05
Ergocryptinine	7.99	576.35	223.09	305.17
Ergotamine	6.97	582.35	208.04	223.08
Ergotaminine	7.24	582.35	223.08	297.10
Ergocristine	7.84	610.35	208.05	223.09
Ergocristinine	8.47	610.35	305.17	223.09

## Supplementary Materials

The following are available online at https://www.mdpi.com/2072-6651/12/11/730/s1, Table S1. Analytical correlations in positive samples; Table S2. Guidelines for interpreting the strength of the correlation (Evans, 1996); Table S3. Performance characteristics of the analytical procedure for the determination of ergot alkaloids.
