# Supplementary material for: Ergot and Ergot Alkaloids in Cereal Grains Intended for Animal Feeding Collected in Slovenia: Occurrence, Pattern and Correlations
Source: Toxins (Basel). 2020 Nov 21;12(11):730. doi: 10.3390/toxins12110730 (PMC7700445; doi:10.3390/toxins12110730)
Supplement: Supplementary file 1 [file toxins-12-00730-s001.pdf]

Supplementary Materials: Ergot and Ergot Alkaloids Intended for Animal Feeding Collected in Slovenia: Occurrence, Pattern And Correlations

Janja Babič, Gabrijela Tavčar-Kalcher, Franci Aco Celar, Katarina Kos, Matjaž Červek and Breda Jakovac-Strajn

Table S1. Analytical correlations in positive samples.

Spearman's rho Correlations		Total EAs	Em	Emn	Es	Esn	Eco	Econ	Ekr	Ekrn	Et	Etn	Ecr	Ecrn
M_{scl} (mg)	r	0.284**	0.228*	0.401**	0.178	0.324**	0.335**	0.325**	0.214*	0.279**	0.252*	0.251*	0.193	0.326**
	p	0.008	0.034	<0.001	0.099	0.002	0.002	0.002	0.047	0.009	0.019	0.019	0.073	0.002
	N	87	87	87	87	87	87	87	87	87	87	87	87	87
M_{scl}/M_{cer} (g/kg)	r	0.315**	0.235*	0.391**	0.194	0.315**	0.356**	0.325**	0.235*	0.313**	0.276**	0.271*	0.230*	0.329**
	p	0.003	0.028	<0.001	0.072	0.003	0.001	0.002	0.029	0.003	0.010	0.011	0.032	0.002
	N	87	87	87	87	87	87	87	87	87	87	87	87	87
Total EAs (μg/kg)	r	1.000	0.558**	0.583**	0.810**	0.734**	0.639**	0.452**	0.630**	0.626**	0.698**	0.686**	0.686**	0.704**
	p		<0.001	<0.001	<0.001	<0.001	<0.001	<0.001	<0.001	<0.001	<0.001	<0.001	<0.001	<0.001
	N	87	87	87	87	87	87	87	87	87	87	87	87	87
Em	r	0.558**	1.000	0.648**	0.500**	0.237*	0.463**	0.325**	0.279**	0.252*	0.469**	0.441**	0.456**	0.435**
	p	<0.001		<0.001	<0.001	0.027	<0.001	0.002	0.009	0.019	<0.001	<0.001	<0.001	<0.001
	N	87	87	87	87	87	87	87	87	87	87	87	87	87
Emn	r	0.583**	0.648**	1.000	0.533**	0.394**	0.499**	0.491**	0.324**	0.348**	0.526**	0.586**	0.445**	0.602**
	p	<0.001	<0.001		<0.001	<0.001	<0.001	<0.001	0.002	0.001	<0.001	<0.001	<0.001	<0.001
	N	87	87	87	87	87	87	87	87	87	87	87	87	87
Es	r	0.810**	0.500**	0.533**	1.000	0.734**	0.621**	0.360**	0.504**	0.414**	0.638**	0.581**	0.689**	0.578**
	p	<0.001	<0.001	<0.001		<0.001	<0.001	0.001	<0.001	<0.001	<0.001	<0.001	<0.001	<0.001
	N	87	87	87	87	87	87	87	87	87	87	87	87	87
Esn	r	0.734**	0.237*	0.394**	0.734**	1.000	0.486**	0.371**	0.403**	0.405**	0.465**	0.473**	0.440**	0.557**
	p	<0.001	0.027	<0.001	<0.001		<0.001	<0.001	<0.001	<0.001	<0.001	<0.001	<0.001	<0.001
	N	87	87	87	87	87	87	87	87	87	87	87	87	87
Eco	r	0.639**	0.463**	0.499**	0.621**	0.486**	1.000	0.740**	0.707**	0.595**	0.521**	0.526**	0.475**	0.361**
	p	<0.001	<0.001	<0.001	<0.001	<0.001		<0.001	<0.001	<0.001	<0.001	<0.001	<0.001	<0.001
	N	87	87	87	87	87	87	87	87	87	87	87	87	87
Econ	r	0.452**	0.325**	0.491**	0.360**	0.371**	0.740**	1.000	0.607**	0.624**	0.285**	0.387**	0,184	0.227*
	p	<0.001	0.002	<0.001	0.001	<0.001	<0.001		<0.001	<0.001	0.007	<0.001	0.089	0.035
	N	87	87	87	87	87	87	87	87	87	87	87	87	87
Ekr	r	0.630**	0.279**	0.324**	0.504**	0.403**	0.707**	0.607**	1.000	0.883**	0.394**	0.420**	0.372**	0.264*
	p	<0.001	0.009	0.002	<0.001	<0.001	<0.001	<0.001		<0.001	<0.001	<0.001	<0.001	0.014
	N	87	87	87	87	87	87	87	87	87	87	87	87	87
Ekrn	r	0.626**	0.252*	0.348**	0.414**	0.405**	0.595**	0.624**	0.883**	1.000	0.289**	0.353**	0.284**	0.309**
	p	<0.001	0.019	0.001	<0.001	<0.001	<0.001	<0.001	<0.001		0.007	0.001	0.008	0.004
	N	87	87	87	87	87	87	87	87	87	87	87	87	87
Et	r	0.698**	0.469**	0.526**	0.638**	0.465**	0.521**	0.285**	0.394**	0.289**	1.000	0.898**	0.683**	0.604**
	p	<0.001	<0.001	<0.001	<0.001	<0.001	<0.001	0.007	<0.001	0.007		<0.001	<0.001	<0.001
	N	87	87	87	87	87	87	87	87	87	87	87	87	87
Etn	r	0.686**	0.441**	0.586**	0.581**	0.473**	0.526**	0.387**	0.420**	0.353**	0.898**	1.000	0.589**	0.618**
	p	<0.001	<0.001	<0.001	<0.001	<0.001	<0.001	<0.001	<0.001	0.001	<0.001		<0.001	<0.001
	N	87	87	87	87	87	87	87	87	87	87	87	87	87
Ecr	r	0.686**	0.456**	0.445**	0.689**	0.440**	0.475**	0,184	0.372**	0.284**	0.683**	0.589**	1.000	0.750**
	p	<0.001	<0.001	<0.001	<0.001	<0.001	<0.001	0.089	<0.001	0.008	<0.001	<0.001		<0.001
	N	87	87	87	87	87	87	87	87	87	87	87	87	87
Ecrn	r	0.704**	0.435**	0.602**	0.578**	0.557**	0.361**	0.227*	0.264*	0.309**	0.604**	0.618**	0.750**	1.000
	p	<0.001	<0.001	<0.001	<0.001	<0.001	0.001	0.035	0.014	0.004	<0.001	<0.001	<0.001	
	N	87	87	87	87	87	87	87	87	87	87	87	87	87

Legend: M—mass, scl—sclerotia, cer—cereals, r—correlation coefficient, p—statistical significance, Sig. (two-tailed). **. Correlation is significant at the 0.01 level (two-tailed); *. Correlation is significant at the 0.05 level (two-tailed). Strength of the positive and negative correlation was evaluated using the Evans (1996) guidelines [1]: a) very weak: 0.00–0.19′ b) weak: 0.20–0.39; c) moderate: 0.40–0.59; d) strong: 0.60–0.79; e) very strong: 0.80–1.0.

Table S2. Guidelines for interpreting the strength of the correlation (Evans, 1996) [1].

Correlation Coefficient Value	Strength
Between ±0.8 and ±1.0	Very strong correlation
Between ±0.6 and ±0.79	Strong correlation
Between ±0.4 and ±0.59	Moderate correlation
Between ±0.2 and ±0.39	Weak correlation
Between ±0.1 and ±0.19	Very weak or no correlation

Table S3. Performance characteristics of the analytical procedure for the determination of ergot alkaloids [2].

	Recovery (%)	RSDr (%)	RSDR (%)
Ergometrine	124	16	19
Ergometrinine	119	16	28
Ergosine	122	8.7	23
Ergosinine	100	12	35
Ergocornine	105	13	12
Ergocorninine	112	6.1	10
Ergocryptine	116	6.2	7.0
Ergocryptinine	103	5.8	13
Ergotamine	118	8.1	13
Ergotaminine	107	6.6	8.0
Ergocristine	109	17	14
Ergocristinine	112	15	12

References

1. Evans, J.D. *Straightforward Statistics for the Behavioral Sciences*; Brooks/Cole Publishing: Pacific Grove, CA, USA, 1996.
2. Topi, D.; Strajn, B.J.; Vrtač, K.P., Kalcher, G.T. Occurrence of ergot alkaloids in wheat from Albania. *Food Addit. Contam. Part A* **2017**, *34*, 1333–1343.
